# Assessment of pleural effusion using intracavitary contrast-enhanced ultrasound: a pilot study

**DOI:** 10.7717/peerj.21765

**Published:** 2026-10-02

**Authors:** Dachuan Tang, Dan Wang, Zhongyu Li, Bohan Lu, Huiming Yi, Wei Zhang

**Affiliations:** Department of Medical Ultrasound, Tongji Hospital, Tongji Medical College, Huazhong University of Science and Technology, Wuhan, China

**Keywords:** Pleural effusion, Intracavitary contrast-enhanced ultrasound, Time-intensity curve quantitative analysis, Exudative pleural effusion, Transudative pleural effusion, Pleural effusion volume quantification

## Abstract

**Objectives:**

To address the limitations of current imaging modalities, the present study aimed to develop and validate a novel intracavitary contrast-enhanced ultrasound (CEUS) approach for the quantitative assessment of pleural effusion (PE) volume and differentiation of exudative PE from transudative PE.

**Methods:**

This cross-sectional diagnostic accuracy study prospectively enrolled 100 patients with PE undergoing US-guided thoracentesis. Intracavitary CEUS was performed using SonoVue microbubbles, and time-intensity curve parameters (A, B, and A+B) were analyzed. PE volume was measured based on the drainage volume, and effusions were classified as exudates or transudates according to Light’s criteria. Diagnostic performance was assessed by receiver operating characteristic (ROC) analysis, and a linear regression model was developed to predict PE volume.

**Results:**

Exudative PE showed significantly higher baseline intensity parameter B (−35.18 ± 5.17 *vs.* −41.41 ± 8.64, *p* < 0.01) and lower peak intensity increment parameter A (1.20 ±  3.27 *vs.* 6.61 ± 6.10, *p* < 0.01) compared to transudative PE. The combined parameter A+B (steady-state microbubble concentration index) showed a strong correlation with PE volume (*r* = 0.912, *R*^2^ = 0.831), yielding the predictive model: V(mL) = −33.345 × (A+B) − 453.071 (intraclass correlation coefficient = 0.950). The ROC analysis revealed moderate diagnostic accuracy for the A value (area under the curve (AUC) = 0.779) and B value (AUC = 0.719) in differentiating exudative PE from transudative PE.

**Conclusion:**

Intracavitary CEUS could serve as a reliable, minimally invasive, feasible radiation-free adjunctive technique for quantifying PE volume and differentiating exudative versus transudative PE in patients undergoing thoracentesis. The A+B-based regression model provides high predictive accuracy, while A and B values facilitate effusion classification. This quantitative CEUS workflow may facilitate clinical decision-making, and its utility for serial monitoring requires further verification in large-scale real-world cohorts.

## Introduction

Pleural effusion (PE) affects 15–20% of hospital inpatients worldwide (Sundaralingam et al., 2024; Munavvar et al., 2025), and arises from diverse disorders, most notably pulmonary infections, malignancy, cardiac failure, and renal disease (Tang, Yi & Zhang, 2024). Since therapeutic strategies and prognosis differ markedly according to etiologies, precise quantitative and qualitative characterization of PE is essential for optimal clinical management.

Chest X-ray, computed tomography (CT), and ultrasound (US) are the most commonly used imaging modalities for evaluating PE in clinical practice, yet each has inherent limitations (Satoto et al., 2022; Zhang et al., 2018; Yalcin-Safak et al., 2017; Ferreiro et al., 2024). Chest X-ray, while widely accessible, has low sensitivity for small-volume effusions and provides no quantitative data. CT delivers precise volumetric assessment and detailed anatomical information but is limited in serial monitoring due to associated ionizing radiation and operational complexity. US is widely recognized as the first-line modality, given its radiation-free nature, bedside applicability, and high reproducibility (Tang, Yi & Zhang, 2024; Scarlata et al., 2020). Nevertheless, conventional grayscale US remains largely qualitative, as it cannot reliably quantify effusion volume and lacks sufficient specificity to distinguish transudates from exudates (Ibitoye et al., 2018; Shiroshita et al., 2020).

In recent years, with the widespread adoption of US microbubble contrast agents, the application of contrast-enhanced ultrasound (CEUS) has gradually expanded to various imaging modalities, including intravascular imaging, cavity imaging, and ductal imaging, yielding favorable clinical outcomes (Jianxiong et al., 2022; Rafailidis et al., 2018; Safai Zadeh et al., 2022). Based on the dynamic distribution data of microbubbles during CEUS, vascular perfusion or intracavitary fluid distribution can be evaluated both intuitively and quantitatively (Yi et al., 2020b). By quantitatively analyzing the distribution of contrast agent within effusions, CEUS holds promise for providing novel approaches to the quantitative and qualitative assessment of PE. To date, only a limited number of studies have investigated intracavitary CEUS for the characterization of pulmonary embolism. Moreover, existing research lacks validated volumetric predictive models, as well as comprehensive head-to-head comparisons with diagnostic metrics derived from conventional ultrasound and CT (Safai Zadeh et al., 2022). Previous studies focused separately on grayscale ultrasound quantification, CT attenuation differentiation, or simple descriptive intracavitary CEUS without integrated quantitative prediction models (Tang, Yi & Zhang, 2024; Yalcin-Safak et al., 2017; Scarlata et al., 2020; Ibitoye et al., 2018; Safai Zadeh et al., 2022).

As a prospective cross-sectional study, this study highlights its incremental advances over existing US, CT, and intracavitary CEUS literature. The present study addresses three key unmet needs in previous studies: (1) it quantifies intracavitary CEUS time-intensity curve (TIC) parameters and correlates such measurements with PE volume and biochemical typing; (2) it constructs and cross-validates a linear regression model for automatic PE volume estimation; (3) it creates a radiation-free, minimally invasive auxiliary assessment scheme exclusive to patients receiving therapeutic thoracentesis.

## Materials and Methods

### Patients

This study was approved by the Ethics committee of Tongji Medical College of Huazhong University of Science and Technology (TJ-IRB202505014) and was performed in compliance with the principles of the World Medical Association Declaration of Helsinki for Biomedical Research involving Human Subjects. A written consent was obtained from each participant.

This study prospectively enrolled participants with PE who underwent US-guided thoracentesis and pleural drainage in Tongji Hospital from December 2024 to May 2025. The inclusion criteria were as follows: (1) age >18 years with stable vital signs and without contraindications to thoracentesis; (2) no thoracic deformity; (3) no history of thoracic surgery; and (4) availability of integrated clinical data. The exclusion criteria were as follows: (1) adverse reactions related to thoracentesis procedures; (2) pleural effusion exceeding 1,000 mL; (3) presence of septation or gas in PE; and (4) poor quality of US images and inaccurate data measurement.

A written informed consent was received from each patient before thoracentesis and intracavitary CEUS, and written informed consent to participate in the study was obtained from all participating patients.

### Ultrasound imaging

A LOGIQ E9 Ultrasound system (GE Healthcare, Little Chalfont, UK), equipped with a 3–5 MHz convex array transducer, was used for US imaging. The system settings during intracavitary CEUS were as follows: depth, 14 cm; transmission frequency, five MHz; mechanical index (MI), 0.14; gain (Gn), 24 dB; and dynamic range (DR), 69 dB. All US data were acquired using the same settings. SonoVue (Bracco, Milan, Italy), microbubbles containing sulfur hexafluoride for injection, was used as the contrast agent in this study. The contrast agent was prepared as follows: five mL of normal saline (0.9% NaCl) was added to reconstitute each bottle of the contrast agent (containing 25 mg of freeze-dried SonoVue); the solution was agitated in accordance with the manufacturer’s instructions.

Patients were placed in a sitting position with natural breathing. Prior to thoracentesis, grayscale ultrasound examinations were performed from top to bottom along the scapular line. The thoracentesis site was identified in an intercostal space with an ultrasound view that clearly demonstrated PE while avoiding the ribs and diaphragm. Each patient was administered a bolus injection of 1.0 mL of SonoVue solution into PE through the thoracentesis site after disinfection and draping. Subsequently, dynamic images of CEUS were recorded for 2 min. A fixed bolus volume of 1.0 mL SonoVue suspension was selected for intracavitary injection for standardized quantification: (1) Preliminary pre-experiment confirmed that 1.0 mL microbubble agent achieves uniform, complete diffusion within pleural fluid within 120 s without excessive echo saturation that distorts time-intensity curve fitting; (2) Uniform injection volume eliminates dose-related differences in microbubble concentration across all subjects, ensuring consistent baseline conditions for linear correlation analysis between A+B parameters and effusion volume; (3) Lower injection volumes produced insufficient contrast signal for reliable region of interest (ROI) measurement, while volumes exceeding 1.0 mL caused microbubble aggregation and signal overflow, invalidating quantitative TIC parameters.

Three ROIs with equal areas were placed at different contrast agent-filled positions where the image quality is clear to develop TICs automatically generated by the dedicated software for the LOGIQ E9 system (Fig. 1). ROIs were created with a fixed diameter of eight mm and uniformly positioned one cm beneath the parietal pleura, while carefully avoiding the pleural lining, sediment, septations, and acoustic artifacts. Quantitative parameters were then generated from the curve fitting formula as follows: F(t) = A(1-exp[-kt])+B, where A is the maximum incremental change, B is the baseline, and t is the time (Yi et al., 2020a). The average parameters values of the three ROIs were recorded, and all measurements were collaboratively performed by two board-certified radiologists (DCT and HMY, with 12 and 15 years of experience, respectively) *via* joint image review to reach a unified conclusion. They were fully blinded to patient clinical data, laboratory results, and final PE typing throughout the measurements.

**Figure 1:**
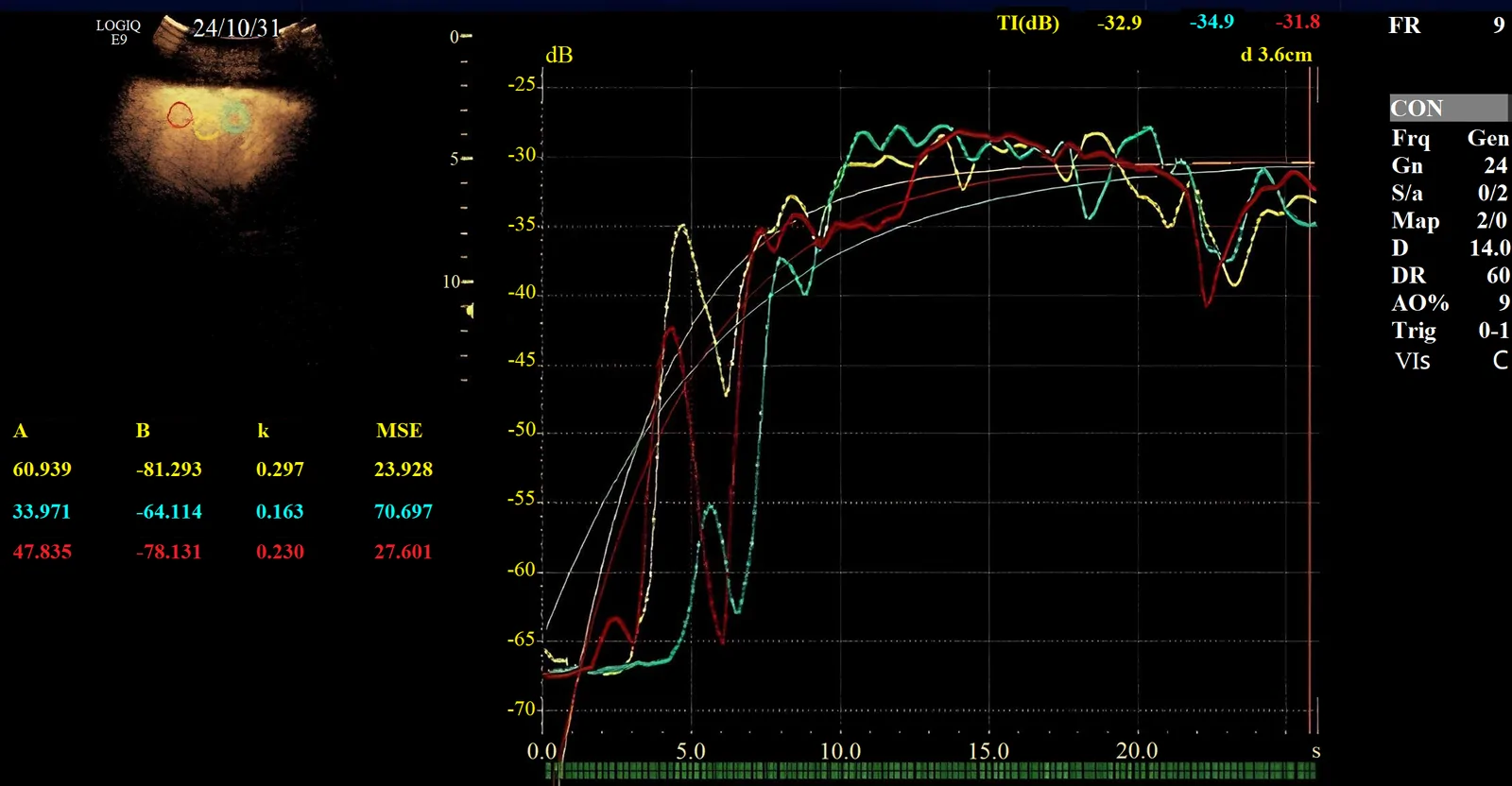
Time Intensive Curve (TIC) for quantitative analysis of contrast-enhanced ultrasound. Three standardized equal-area ROIs were placed in artifact-free contrast-filled pleural fluid regions to generate fitted TIC curves and extract quantitative A, B, and A+B parameters.

### Thoracentesis and drainage

Following CEUS, US-guided thoracentesis and catheter drainage of the PE were performed. A 7F pigtail catheter (YB-A-II 2.3/235, Jiangsu Yubang Medical Equipment Technology Co., Ltd., Taizhou, Jiangsu, China) was indwelled into the thoracic cavity through the Seldinger catheterization procedure, and a sterile bag was then attached for drainage. Complete drainage was recognized based on the absence of an anechoic area between the visceral and parietal pleura on US imaging. The total amount of drainage was recorded as the actual volume of PE when complete drainage was achieved. If the drainage volume reached 800 mL and US still showed the presence of effusion in the pleural cavity, the drainage was suspended during the study.

### PE biochemical typing

The PE and serum samples of all patients were subjected to the following biochemical tests: total protein (TP) and lactate dehydrogenase (LDH). The following Light’s criteria were considered (Gardiner et al., 2024): (1) the ratio of PE TP to serum TP > 0.5; (2) the ratio of PE LDH to serum LDH > 0.6; and (3) PE LDH >  two-thirds of the upper limit of the normal serum LDH value. If one or more of the abovementioned criteria were met, the effusion was recognized as an exudate based on the patient’s medical history and other clinical data; otherwise, the effusion was identified as a transudate.

### Statistical analysis

All statistical analyses were performed using MedCal software (Epic Systems Corporation, Verona, WI, USA), with two-sided *p* < 0.05 defined as statistically significant. Continuous variables are presented as mean ± standard deviation (SD). Normality of distribution was assessed *via* Shapiro–Wilk test; Student’s *t*-test was used for normally distributed data, while Mann–Whitney *U* nonparametric testing was applied for non-normal distributions. Multiple CEUS parameters (A, B, A+B) were analyzed simultaneously; Bonferroni correction was applied to adjust for multiple comparison bias to control type I error. ROC curve analysis quantified diagnostic performance of A and B for PE subtyping; maximum Youden index determined optimal cut-off values. All AUC results are reported with 95% confidence intervals.

Pearson linear correlation evaluated associations between CEUS parameters and reference drainage volume. Based on the correlation coefficient values, a linear regression model was constructed with drainage volume (V) as the dependent variable and CEUS parameters as the independent variable. To avoid internal overfitting from single-dataset validation, the full cohort (*n* = 100) was randomly split into training set (70%) and hold-out validation set (30%) for cross-validation of the regression model.

Bland-Altman plots and intraclass correlation coefficient (ICC) analyses were used to confirm the correlations between PE volume derived from the generated linear model and the actual drainage volume.

### Sample size justification

As a pilot exploratory study, we completed pre-enrollment power analysis with preset parameters (expected AUC = 0.75, *α* = 0.05, power = 0.80), indicating a minimal sample of 96 patients. A total of 100 consecutive participants were enrolled and equally allocated to exudative (*n* = 50) and transudative (*n* = 50) subgroups to simplify intergroup comparisons. We recognize this artificially balanced ratio diverges from their natural clinical prevalence; subgroup positive predictive values calibrated to real-world PE prevalence are reported in the ‘Discussion’.

## Results

A total of 100 participants (58 males, 42 females; mean age 56.6 ± 13.1 years, mean PE volume 693.5 ± 160.0 mL) were enrolled in this study. Of these, 50 had exudative PE and 50 had transudative PE (Fig. 2). All patients safely underwent intracavitary CEUS without any side effects.

**Figure 2:**
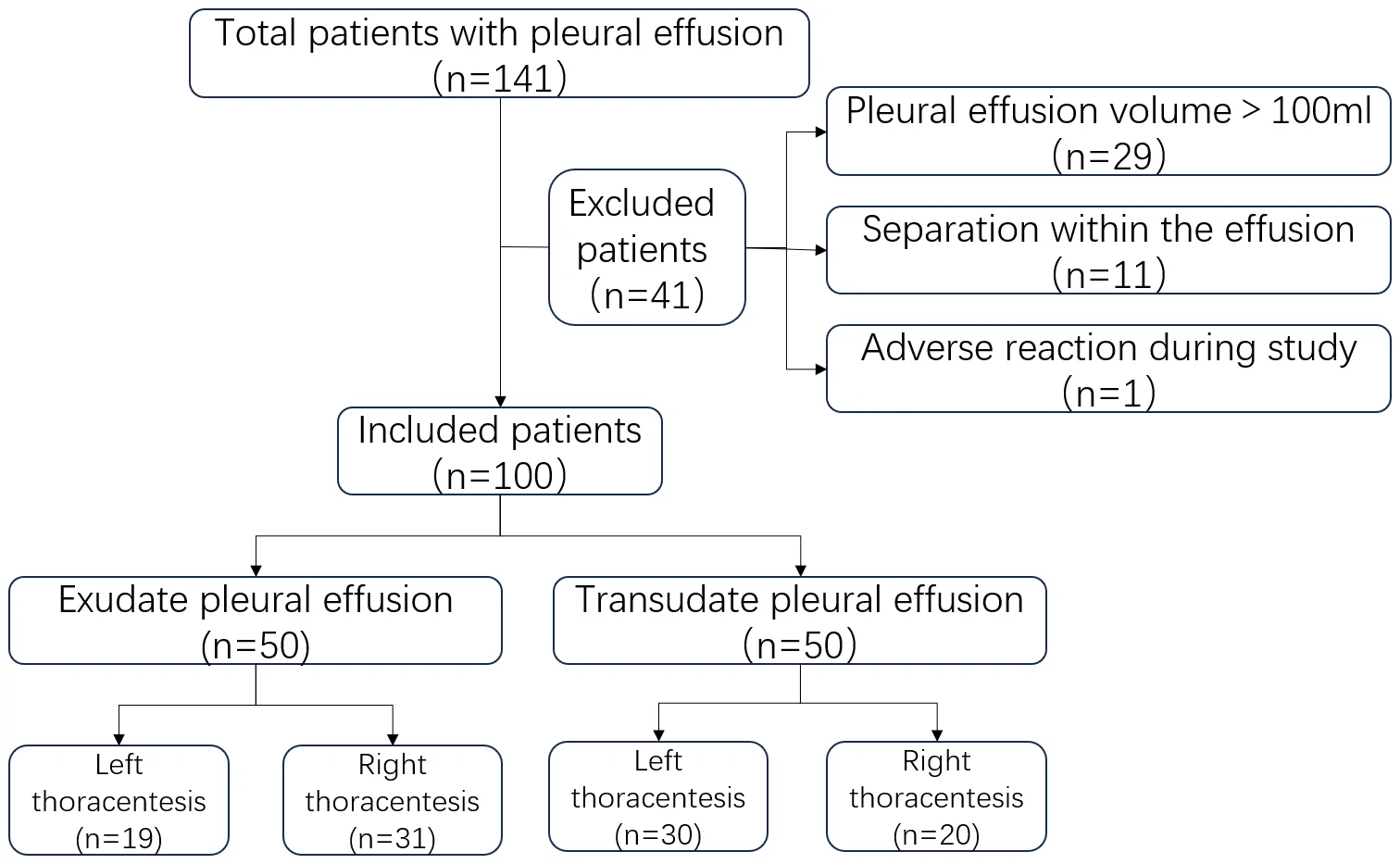
Flowchart of patient enrollment. This study enrolled a stratified sample of 50 exudative and 50 transudative pleural effusion patients.

Baseline clinical characteristics: There were no statistically significant intergroup differences in terms of age, sex distribution, or total PE drainage volume; however, etiological distribution (malignancy, infection, heart failure, polyserous effusion) differed substantially between exudative and transudative subgroups (Table 1).

**Table 1:** Clinical characteristics of patients included in this study.

	Exudate (*n* = 50)	Transudate (*n* = 50)	*p* value
Age (y)	59.1 ± 10.6	54.1 ± 14.9	0.06
Male (%)	31 (62%)	24 (48%)	0.16
PE volume (ml)	701.4 ± 146.5	685.7 ± 173.7	0.63
TP	42.9 ± 7.7	19.7 ± 10.7	<0.01
LDH	430.9 ± 201.9	50.9 ± 19.7	<0.01
Location			Total
Left thoracentesis	19 (38%)	30 (60%)	49 (49%)
Right thoracentesis	31 (62%)	20 (40%)	51 (51%)
Etiology			
Tumor-related	37 (74%)	5 (10%)	42 (42%)
Infection-related	13 (26%)	0	13 (13%)
Polyserous effusion	0	36 (72%)	36 (36%)
Heart failure	0	9 (18%)	9 (9%)

### Comparison of CEUS quantitative parameters between exudative PE and transudative PE

Exudative PE showed a significantly higher B value (−35.18 ± 5.17 *vs.* −41.41 ± 8.64, *p* < 0.01) and a lower A value (1.20 ± 3.27 *vs.* 6.61 ± 6.10, *p* < 0.01) compared to transudative PE. However, exudative PE and transudative PE showed no significant difference in the A+B value (−33.97 ± 3.48 *vs.* −34.80 ±  5.09, *p* = 0.34) (Fig. 3).

**Figure 3:**
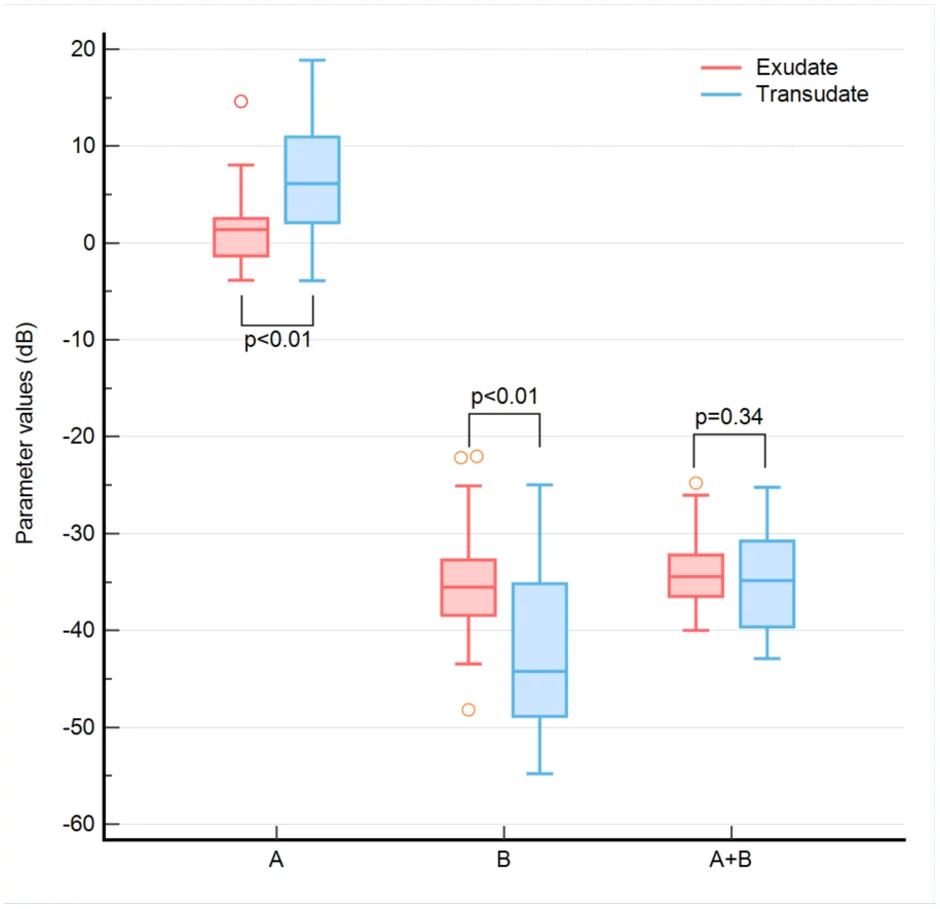
Comparison of CEUS quantitative TIC parameters A, B, and A+B between exudative and transudative pleural effusion subgroups. Exudative pleural effusion showed significantly higher B value, lower A value and no significant difference in the A+B value compared to transudative pleural effusion.

### Diagnostic value of CEUS quantitative parameters A and B for differentiating exudative PE and transudative PE

The ROC analyses showed that both A and B values could differentiate exudative PE from transudative PE, with AUC of 0.779 and 0.719, respectively. For exudative PE diagnosis, the optimal cutoff value of parameter A was 3.905, with sensitivity and specificity of 90.0% and 66.0%, respectively. Additionally, a Parameter B value ≥ −43.396 suggested an exudative PE diagnosis, with a sensitivity of 96.0% and a specificity of 54.0%, respectively. The low specificity of parameter B indicates nearly half of transudative effusions will be misclassified as exudative (Fig. 4).

**Figure 4:**
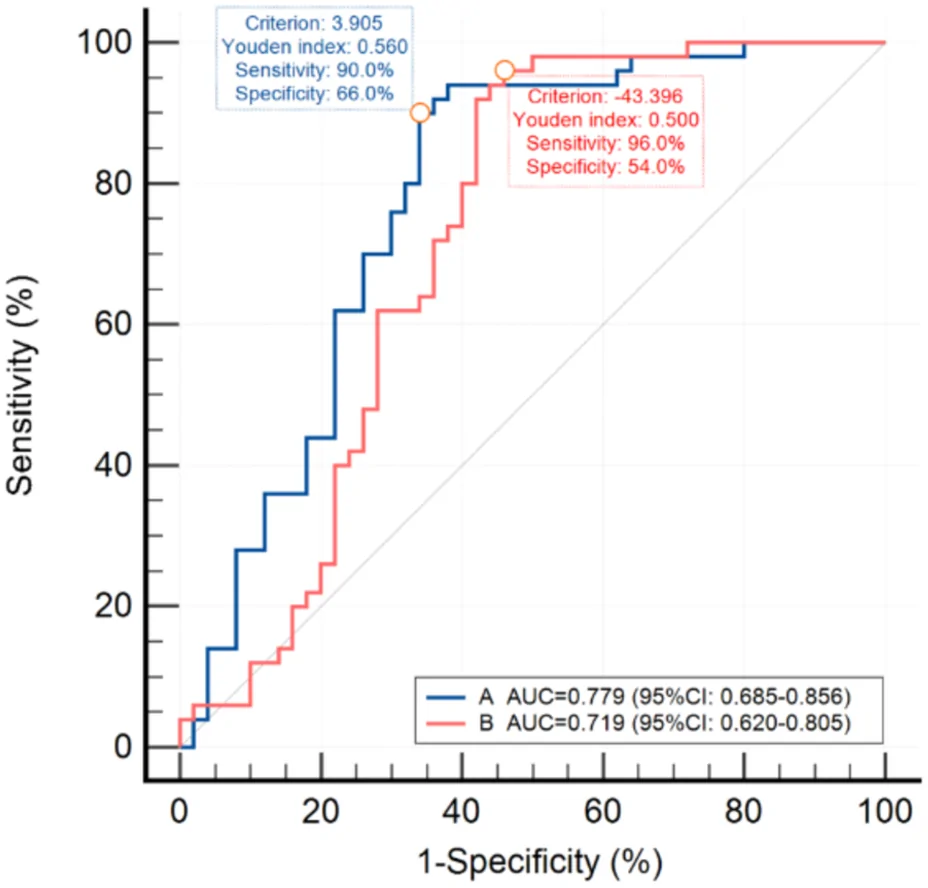
ROC analyses to differentiate exudative PE from transudative PE. Both parameter A and B could differentiate exudative pleural effusion from transudative pleural effusion, with AUC values of 0.779 and 0.719, respectively. Cut-off thresholds, sensitivity, and specificity for each parameter are annotated.

### Analysis between CEUS quantitative parameters and the drainage volume of PE

A scatterplot of drainage volume and CEUS quantitative parameter A+B was created. A significant negative correlation was observed between the A+B value and the drainage volume (*p* < 0.01; correlation coefficient *r* = 0.912, coefficient of determination *R*^2^ = 0.831). By using the drainage V as the dependent variable and the A+B value as the independent variable, a univariate linear regression model was constructed as follows: V (mL) = −33.345 × (A + B) − 453.071 (Fig. 5).

**Figure 5:**
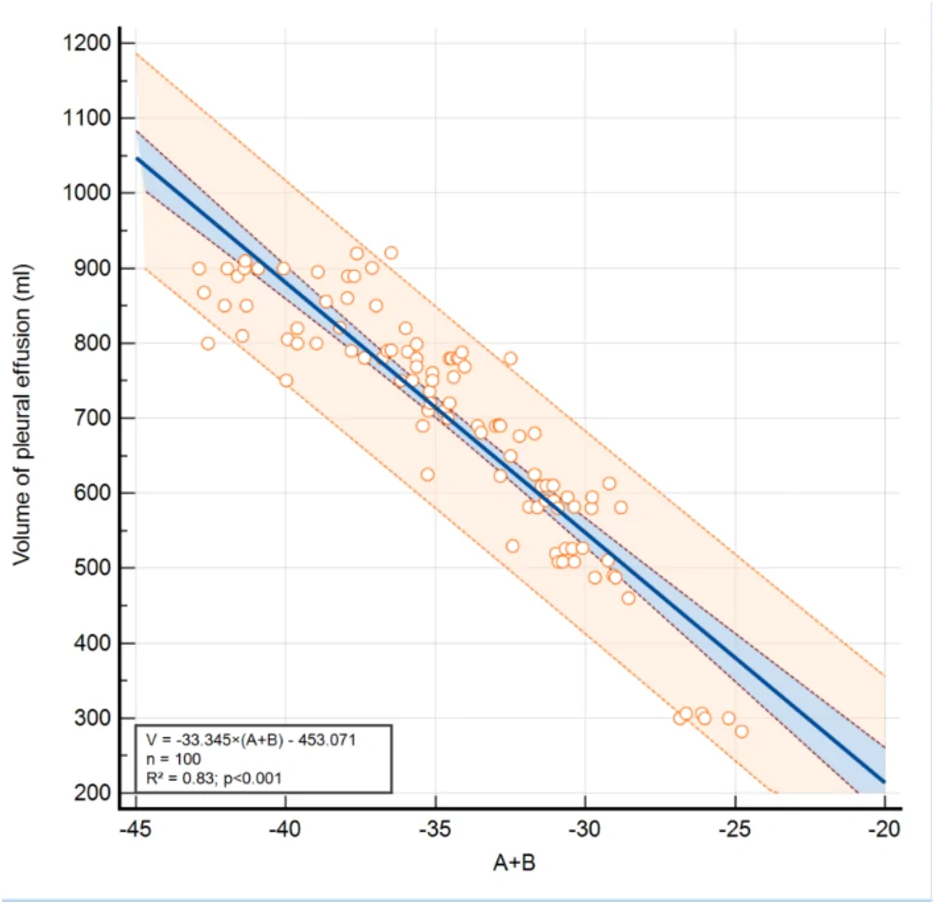
Scatterplot of the correlation between the A+B value and the drainage volume of pleural effusion. A significant negative correlation existed between the A+B value and the drainage volume.

### Cross-validated model consistency

After split-sample hold-out cross-validation, Bland-Altman plotting demonstrated that 95% of differences between predicted volume (Vprediction) and real drainage volume (Vreal) fell within the 95% limits of agreement (Fig. 6). The overall ICC between Vprediction and Vreal was 0.950 (95% CI [0.925–0.966], *p* < 0.01), confirming excellent predictive reliability of the regression model.

**Figure 6:**
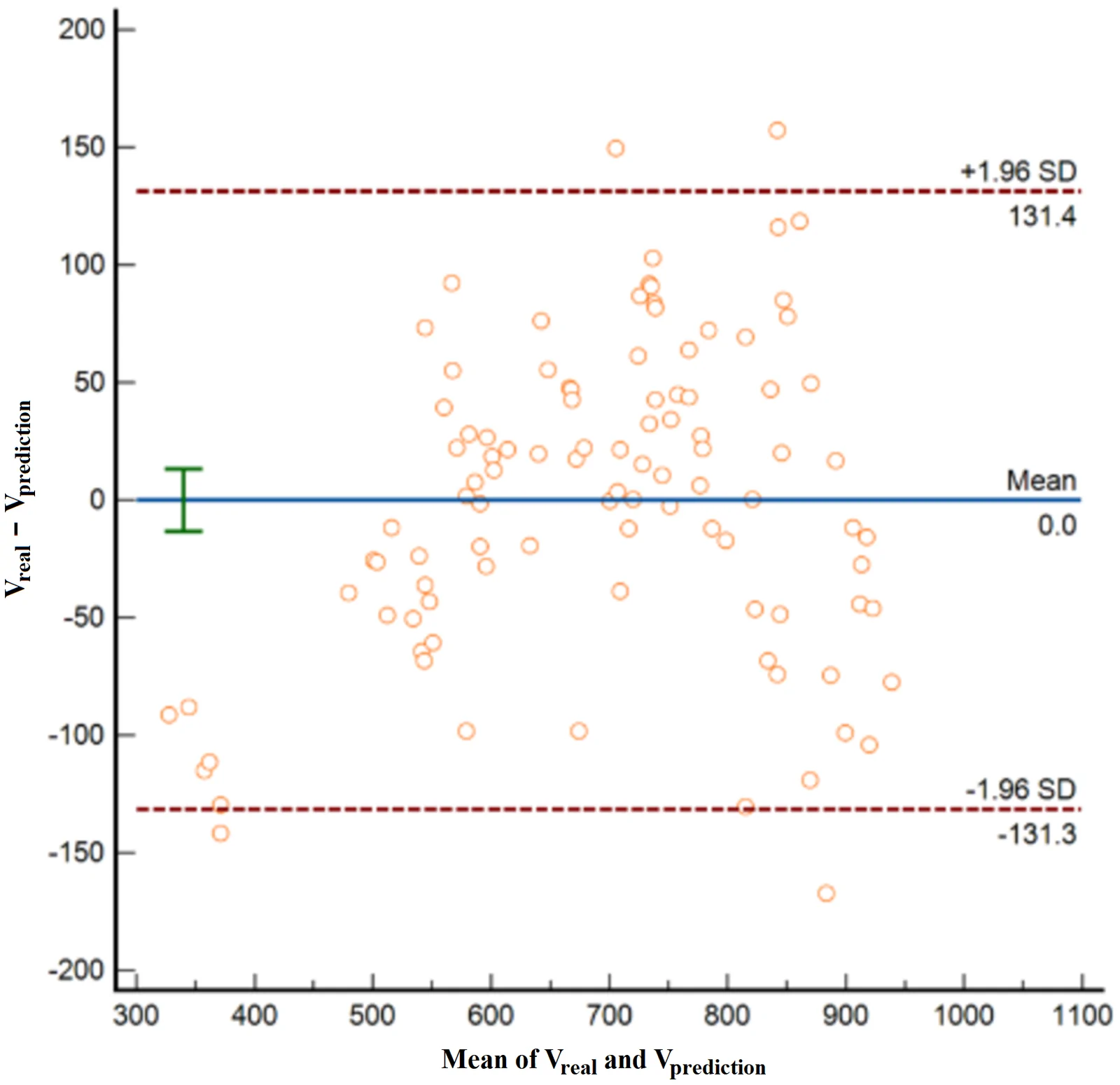
A Bland-Altman plot comparing Vprediction and Vreal. The prediction results of the developed model exhibited good consistency with the actual drainage volume.

### Prediction accuracy of the regression model for PE volume

The Vpredictions were calculated using the constructed regression model mentioned above, which was then subjected to a consistency test against the Vreals among participants in this study. A Bland-Altman plot was generated to compare Vprediction and Vreal, and found that most differences between the two variables fell within the 95% CI, indicating the consistency between Vprediction and Vreal (Fig. 6). ICC between Vprediction and Vreal was 0.950 (95% confidence interval CI [0.925–0.966], *p* < 0.01), further confirming that the model has good reliability for predicting PE volume.

## Discussion

CEUS is widely used in clinical practice nowadays, owing to its advantages of real-time dynamic imaging, lack of radiation, and simple operational procedures (Yi et al., 2020a). Ultrasound contrast agents are microbubbles similar in size to red blood cells, with strong echogenic signals (Sidhu et al., 2018). Typically, after intravenous injection of these microbubbles, tissue perfusion and vascular architecture can be evaluated by detecting and analyzing changes in microbubble echo intensity (Wang et al., 2023). In addition, when contrast agents are injected into body cavities *in vivo*, the visualization of microbubbles allows assessment of the extent and morphology of the cavities (Yi et al., 2020b; Sidhu et al., 2018). In the present study, microbubbles are utilized for CEUS imaging of PE *via* an intracavitary approach, and quantitative analyses were performed to assess its feasibility in differentiating exudative from transudative PE and quantifying PE volume. The findings of this study demonstrated that intracavitary CEUS is highly suitable for the evaluation of PE.

To date, numerous studies have been conducted to assess the quality of PE using imaging modalities. However, to the best of our knowledge, only a limited number of studies have reported on the feasibility of US imaging for the differential diagnosis of PE quality (Yalcin-Safak et al., 2017; Tang & Zhang, 2024). Our prior research demonstrated that the echo intensity (EI) value derived from grayscale US had diagnostic utility for distinguishing exudative from transudative PE (AUC = 0.648) (Zhang & Tang, 2024). In the present study, parameters generated from intracavitary CEUS were used to evaluate PE, revealing that both Parameter A and Parameter B can differentiate exudative from transudative PE, with AUC values of 0.779 and 0.719, respectively. These findings suggested that intracavitary CEUS may offer superior performance to grayscale US in assessing PE quality. Furthermore, intracavitary CEUS exhibited comparable performance to CT-based methods for the differential diagnosis of PE quality, with a reported AUC of 0.775 (Yalcin-Safak et al., 2017). This study uniquely provides a validated volumetric prediction model absent from all prior intracavitary CEUS PE literature, representing the core novel contribution of this work (Safai Zadeh et al., 2022).

The present study illustrated that exudative PE exhibited a significantly lower A value and a significantly higher B value compared with transudative PE. Transudative PE arises primarily from increased vascular hydrostatic pressure or reduced intravascular colloid osmotic pressure, seen in conditions like superior vena cava obstruction, constrictive pericarditis, hypoproteinemia, *etc*. Exudative PE, by contrast, stems from increased pleural permeability or impaired parietal pleural lymphatic drainage, common in pulmonary/pleural infections and thoracic tumors (Ferreiro et al., 2024). Consequently, exudative PE has more complex and diverse biochemical components than transudative PE, with higher levels of TP, LDH, cholesterol, and prostaglandin E (Huggins, 2010; Pace et al., 2006). Following the injection of US contrast agent into the PE, echo intensity increases as microbubbles diffuse. Based on TIC fitting, parameter B represented the baseline echo intensity of PE, whereas parameter A reflected the maximum incremental echo intensity (Yi et al., 2020a). Accordingly, resulting from more complex biochemical components, exudative PE exhibited significantly higher baseline echo intensity (the B value) than transudative PE (Tang & Zhang, 2024). However, in exudative PE, these complex biochemical components increased adsorption to and disruption of microbubbles (Martynov et al., 2011), leading to a significantly lower A value compared to transudative PE.

Over time, the contrast agent concentration plateaued following complete diffusion and convection. During this phase, the contrast agent was relatively evenly distributed within the effusion, and the A+B value reflected the microbubble concentration in a stable state. Since exudative PE exhibited a higher B value and a lower A value compared with transudative PE, no significant difference existed in terms of the A+B value between the two groups in this study. This result indicated that the A+B value could not be affected by the quality of PE during intracavitary CEUS.

Furthermore, a strong correlation between the A+B value with PE volume was revealed in this study. Since the contrast agent injection dosage was consistent across all cases, variations in effusion volume resulted in differences in the final concentration of the contrast agent, which in turn led to variability in the A+B values derived from CEUS. Accordingly, this study established a linear regression model by using the A+B value as the independent variable and PE drainage volume as the dependent variable, and this model exhibited excellent reliability in predicting PE volume.

This cohort included exactly 50 exudative and 50 transudative effusions, creating an artificially balanced 1:1 ratio inconsistent with real-world clinical prevalence, where transudative effusions account for approximately 60–70% of all hospitalized PE cases. Using standard population prevalence estimates, we calculated adjusted positive predictive values (PPV): at 65% transudate prevalence, parameter B’s PPV falls to ∼61%, further limiting its standalone clinical utility due to low specificity (54%). Parameter A delivers more balanced PPV (∼78%) under natural prevalence, making it more suitable for routine subtype screening. The clinical trade-off between high sensitivity (B) and balanced specificity (A) is discussed to contextualize moderate overall diagnostic accuracy (AUC 0.70–0.78).

Volumetric quantification advantages and limitations of complete drainage criteria Researchers have demonstrated that manually outlining the PE contour layer-by-layer on CT images and subsequently calculating volume *via* software yielded relatively accurate PE quantification (Yalcin-Safak et al., 2017). However, this method is unsuitable for PE monitoring due to its cumbersome process, susceptibility to human error, and the ionizing radiation associated with CT. Additionally, our prior study utilized manual layer-by-layer contouring in ultrasound imaging to calculate PE volume. This approach avoided radiation exposure and yielded favorable results but remained time-consuming and prone to operator error (Tang, Yi & Zhang, 2024). In contrast, the intracavitary CEUS quantitative parameter-based method developed in this study offered key advantages: it eliminated repeated manual measurements, enabled automatic image analysis, and allowed rapid calculation of PE volume after ROI selection.

This study had several limitations. First, CEUS quantitative parameters were susceptible to variations in equipment and imaging settings; thus, the results in this study were limited to the specific instruments and software used herein. Second, the sample size of this study was relatively small, highlighting the need for multi-center larger clinical cohorts to further validate and facilitate its application. Third, subgroup analyses of PE stratified by underlying etiologies were not conducted. To enhance diagnostic accuracy, future studies should investigate differences in CEUS quantitative parameters among PEs of varying etiologies. Fourth, Ultrasound-verified full drainage was adopted as the reference standard in this study, yet this metric contains inherent limitations. Small amounts of residual fluid can be trapped in pleural fissures despite apparent complete drainage, which slightly underestimates actual total PE volume. This inherent bias should be considered when interpreting the predictive performance of our model. Fifth, patients with PE volumes exceeding 1,000 mL were excluded from the present study, which restricts the direct clinical generalizability of our findings to large-volume effusions. Future investigations will eliminate this volume-based exclusion cutoff. Sixth, drainage was suspended once cumulative fluid reached 800 mL even if ultrasonography still showed residual pleural effusion. This arbitrary stopping threshold leads to systematic underestimation of total true effusion volume in patients with large effusions, introducing measurement bias into the linear regression model. Together with the exclusion of patients with effusion >1,000 mL, this two-fold volume restriction limits the generalizability of our volumetric prediction model for patients with massive pleural effusion. Future work will remove both the 800 mL drainage pause cutoff and 1,000 mL enrollment exclusion to validate the model across the full spectrum of effusion volumes. Seventh, all quantitative TIC measurements were completed *via* joint consensus review by two radiologists rather than separate independent blinded reading. This joint reading design eliminates inter-observer measurement variability but cannot generate inter-rater ICC values to quantify individual reader disagreement, which limits external reproducibility reference for other ultrasound centers with single-operator workflows. Subsequent validation trials will implement independent duplicate blinded measurements to calculate inter-observer ICC for A, B and A+B parameters.

## Conclusion

This pilot cross-sectional study demonstrated that intracavitary CEUS could serve as a reliable, minimally invasive, radiation-free adjunctive method to quantify PE volume and differentiate exudative PE from transudative PE. The A+B-based regression model provided high predictive accuracy, while parameters A and B facilitated PE classification. The quantitative intracavitary CEUS protocol may support clinical decision-making for PE management, yet its performance for repeated serial clinical monitoring requires further large-cohort prospective verification. Intracavitary CEUS may enhance clinical decision-making.

##  Supplemental Information

10.7717/peerj.21765/supp-1 Supplemental Information 1 Raw data

10.7717/peerj.21765/supp-2 Supplemental Information 2 STROBE checklist

## Additional Information and Declarations

### Data Availability

The following information was supplied regarding data availability: The raw measurements are available in the Supplementary Files.
