# Supplementary material for: Assessment of pleural effusion using intracavitary contrast-enhanced ultrasound: a pilot study
Source: PeerJ. 2026 Oct 2;14:e21765. doi: 10.7717/peerj.21765 (PMC13637638; doi:10.7717/peerj.21765)
Supplement: Supplementary file 1 [file peerj-14-21765-s002.docx]

STROBE Statement—checklist of items that should be included in reports of observational studies

|  | Item No. | Recommendation | Page  No. | Relevant text from manuscript |
| --- | --- | --- | --- | --- |
| Title and abstract | 1 | (a) Indicate the study’s design with a commonly used term in the title or the abstract | 2 | This cross-sectional diagnostic accuracy study prospectively enrolled 100 patients with PE undergoing US-guided thoracentesis. |
|  |  | (b) Provide in the abstract an informative and balanced summary of what was done and what was found | 2 | Intracavitary CEUS was performed using SonoVue microbubbles, and time-intensity curve parameters (A, B, and A+B) were analyzed. Intracavitary CEUS could serve as a reliable, minimally invasive, feasible radiation-free adjunctive technique for quantifying PE volume and differentiating exudative versus transudative PE in patients undergoing thoracentesis. |
| Introduction | | | |  |
| Background/rationale | 2 | Explain the scientific background and rationale for the investigation being reported | 3 | Conventional grayscale US remains largely qualitative, as it cannot reliably quantify effusion volume and lacks sufficient specificity to distinguish transudates from exudates.  Based on the dynamic distribution data of microbubbles during CEUS, vascular perfusion or intracavitary fluid distribution can be evaluated both intuitively and quantitatively. |
| Objectives | 3 | State specific objectives, including any prespecified hypotheses | 4 | CEUS holds promise for providing novel approaches to the quantitative and qualitative assessment of PE. |
| Methods | | | |  |
| Study design | 4 | Present key elements of study design early in the paper | 4 | The present study addresses three key unmet needs in previous studies: (1) it quantifies intracavitary CEUS TIC parameters and correlates such measurements with PE volume and biochemical typing; (2) it constructs and cross-validates a linear regression model for automatic PE volume estimation; (3) it creates a radiation-free, minimally invasive auxiliary assessment scheme exclusive to patients receiving therapeutic thoracentesis. |
| Setting | 5 | Describe the setting, locations, and relevant dates, including periods of recruitment, exposure, follow-up, and data collection | 5 | This study prospectively enrolled participants with PE who underwent US-guided thoracentesis and pleural drainage in Tongji Hospital from December 2024 to May 2025. |
| Participants | 6 | (a) Cohort study—Give the eligibility criteria, and the sources and methods of selection of participants. Describe methods of follow-up  Case-control study—Give the eligibility criteria, and the sources and methods of case ascertainment and control selection. Give the rationale for the choice of cases and controls  Cross-sectional study—Give the eligibility criteria, and the sources and methods of selection of participants | 9 | A total of 100 consecutive participants were enrolled and equally allocated to exudative (n = 50) and transudative (n = 50) subgroups to simplify intergroup comparisons. |
|  |  | (b) Cohort study—For matched studies, give matching criteria and number of exposed and unexposed  Case-control study—For matched studies, give matching criteria and the number of controls per case | N/A |  |
| Variables | 7 | Clearly define all outcomes, exposures, predictors, potential confounders, and effect modifiers. Give diagnostic criteria, if applicable | 8 | If one or more of the abovementioned criteria were met, the effusion was recognized as an exudate based on the patient’s medical history and other clinical data; otherwise, the effusion was identified as a transudate. |
| Data sources/ measurement | 8* | For each variable of interest, give sources of data and details of methods of assessment (measurement). Describe comparability of assessment methods if there is more than one group | 7 | Quantitative parameters were then generated from the curve fitting formula as follows: F(t) = A(1-exp[-kt])+B, where A is the maximum incremental change, B is the baseline, and t is the time. |
| Bias | 9 | Describe any efforts to address potential sources of bias | 7 | The average parameters values of the three ROIs were recorded, and all measurements were collaboratively performed by two board-certified radiologists (DCT and HMY, with 12 and 15 years of experience, respectively) via joint image review to reach a unified conclusion. |
| Study size | 10 | Explain how the study size was arrived at | 5 | This study prospectively enrolled participants with PE who underwent US-guided thoracentesis and pleural drainage in Tongji Hospital from December 2024 to May 2025. |

Continued on next page

| Quantitative variables | 11 | Explain how quantitative variables were handled in the analyses. If applicable, describe which groupings were chosen and why | 7 | The following Light’s criteria were considered: (1) the ratio of PE TP to serum TP > 0.5; (2) the ratio of PE LDH to serum LDH > 0.6; and (3) PE LDH > two-thirds of the upper limit of the normal serum LDH value. |
| --- | --- | --- | --- | --- |
| Statistical methods | 12 | (a) Describe all statistical methods, including those used to control for confounding | 8 | All statistical analyses were performed using MedCal software, with two-sided p < 0.05 defined as statistically significant. |
|  |  | (b) Describe any methods used to examine subgroups and interactions | N/A |  |
|  |  | (c) Explain how missing data were addressed | N/A |  |
|  |  | (d) Cohort study—If applicable, explain how loss to follow-up was addressed  Case-control study—If applicable, explain how matching of cases and controls was addressed  Cross-sectional study—If applicable, describe analytical methods taking account of sampling strategy | 9 | Sample size justification |
|  |  | (e) Describe any sensitivity analyses | N/A |  |
| Results | | | | |
| Participants | 13* | (a) Report numbers of individuals at each stage of study—eg numbers potentially eligible, examined for eligibility, confirmed eligible, included in the study, completing follow-up, and analysed | 9 | Of these, 50 had exudative PE and 50 had transudative PE (figure 2). All patients safely underwent intracavitary CEUS without any side effects.  There were no statistically significant intergroup differences in terms of age, sex distribution, or total PE drainage volume; however, etiological distribution (malignancy, infection, heart failure, polyserous effusion) differed substantially between exudative and transudative subgroups (Table 1). |
|  |  | (b) Give reasons for non-participation at each stage | N/A |  |
|  |  | (c) Consider use of a flow diagram | N/A |  |
| Descriptive data | 14* | (a) Give characteristics of study participants (eg demographic, clinical, social) and information on exposures and potential confounders | 9 | Of these, 50 had exudative PE and 50 had transudative PE (figure 2). All patients safely underwent intracavitary CEUS without any side effects.  There were no statistically significant intergroup differences in terms of age, sex distribution, or total PE drainage volume; however, etiological distribution (malignancy, infection, heart failure, polyserous effusion) differed substantially between exudative and transudative subgroups (Table 1). |
|  |  | (b) Indicate number of participants with missing data for each variable of interest | N/A |  |
|  |  | (c) *Cohort study*—Summarise follow-up time (eg, average and total amount) | N/A |  |
| Outcome data | 15* | *Cohort study*—Report numbers of outcome events or summary measures over time | N/A |  |
|  |  | *Case-control study—*Report numbers in each exposure category, or summary measures of exposure | N/A |  |
|  |  | *Cross-sectional study—*Report numbers of outcome events or summary measures | 9 | Of these, 50 had exudative PE and 50 had transudative PE (figure 2). All patients safely underwent intracavitary CEUS without any side effects.  There were no statistically significant intergroup differences in terms of age, sex distribution, or total PE drainage volume; however, etiological distribution (malignancy, infection, heart failure, polyserous effusion) differed substantially between exudative and transudative subgroups (Table 1). |
| Main results | 16 | (*a*) Give unadjusted estimates and, if applicable, confounder-adjusted estimates and their precision (eg, 95% confidence interval). Make clear which confounders were adjusted for and why they were included | 9-10 | Comparison of CEUS quantitative parameters between exudative PE and transudative PE  Diagnostic value of CEUS quantitative parameters A and B for differentiating exudative PE and transudative PE Analysis between CEUS quantitative parameters and the drainage volume of PE |
|  |  | (*b*) Report category boundaries when continuous variables were categorized | N/A |  |
|  |  | (*c*) If relevant, consider translating estimates of relative risk into absolute risk for a meaningful time period | N/A |  |

Continued on next page

| Other analyses | 17 | Report other analyses done—eg analyses of subgroups and interactions, and sensitivity analyses | 11 | Prediction accuracy of the regression model for PE volume |
| --- | --- | --- | --- | --- |
| Discussion | | | | |
| Key results | 18 | Summarise key results with reference to study objectives | 11-15 | discussion |
| Limitations | 19 | Discuss limitations of the study, taking into account sources of potential bias or imprecision. Discuss both direction and magnitude of any potential bias | 15-16 | This study had several limitations. |
| Interpretation | 20 | Give a cautious overall interpretation of results considering objectives, limitations, multiplicity of analyses, results from similar studies, and other relevant evidence | 15 | the results in this study were limited to the specific instruments and software used herein. |
| Generalisability | 21 | Discuss the generalisability (external validity) of the study results | 16-17 | conclusion |
| Other information | |  | | |
| Funding | 22 | Give the source of funding and the role of the funders for the present study and, if applicable, for the original study on which the present article is based | N/A |  |

*Give information separately for cases and controls in case-control studies and, if applicable, for exposed and unexposed groups in cohort and cross-sectional studies.

**Note:** An Explanation and Elaboration article discusses each checklist item and gives methodological background and published examples of transparent reporting. The STROBE checklist is best used in conjunction with this article (freely available on the Web sites of PLoS Medicine at http://www.plosmedicine.org/, Annals of Internal Medicine at http://www.annals.org/, and Epidemiology at http://www.epidem.com/). Information on the STROBE Initiative is available at www.strobe-statement.org.
